# Editorial: Cancer Epidemiology in China: What We Have Learnt So Far?

**DOI:** 10.3389/fonc.2020.00106

**Published:** 2020-02-07

**Authors:** Tianhui Chen, Xiaochen Shu, Hao Liu, Jianguang Ji

**Affiliations:** ^1^Department of Cancer Prevention, Cancer Hospital of the University of Chinese Academy of Sciences, Zhejiang Cancer Hospital, Hangzhou, China; ^2^School of Public Health, Soochow University, Suzhou, China; ^3^Nanfang Hospital, Southern Medical University, Guangzhou, China; ^4^Center for Primary Health Care Research, Lund University, Lund, Sweden

**Keywords:** cancer epidemiology, China, cancer incidence, cancer mortality, cancer survival, cohort

Cancer epidemiology has developed greatly during the past decades in China. Overall 36 cancer registers in China were included by the International Agency for Research on Cancer and the International Association of Cancer Registries for their joint publication “Cancer Incidence in Five Continents” (Volume XI) which provides the reference source of data on the incidence of cancer in China. Many scientists have been successfully recruited from comprehensive research institutions, and accelerate the development of cancer epidemiology in China. In this Research Topic, we have received a total of 36 submissions. We selected 16 articles contributed by 216 authors, which have received 24,262 views and nearly 4,000 downloads so far. Our collection covers different types of cancer and various study designs.

Using colorectal cancer (CRC) incidence data from the Cancer Incidence in Five Continents, Volume XI dataset and the age-standardized incidence rate and age-standardized mortality rate of CRC from the 2016 Global Burden of Diseases Study, Dr. Tianjiang Ma's group found a steady increase in the CRC incidence in China over the past three decades and predicted a further increase in the near future (Zhang et al.). Using data from local cancer register in Yangzhou city, the incidence and mortality rates of esophageal and gastric cancers showed a downward trend whereas CRC was on the rise as a whole, suggesting heterogeneous risk factors for these common digestive cancers in China (Shao et al.). Another study leaded by Profs Maode Lai and Yimin Zhu's group found the prediction of CRC prognosis might be improved by using informative differentially expressed gene (DEG) compared to that using the TNM staging system (Pan et al.). As for CRC cancer screening, Wu et al. found that cutoff points of risk score should be optimized and stool-based test should be improved for large-scale usage in Chinese population.

The contribution of infectious microbes on cancer might be more predominant in developing countries such as China. High prevalence of HPV-16 was found to be associated with the incidence of head and neck cancers, as evidenced by a recent meta-analysis using publications on Chinese population during 2006–2018 (Guo et al.). Prof. Guangwen Cao's group found HBV promotes the aggressiveness of primary liver cancer in Chinese population, and the contributions of HBV to intrahepatic cholangiocarcinoma and other etiological factors to HCC might be indirect via arousing non-resolving inflammation (Yang et al.). However, another study found no correlation between Epstein-Barr virus (EBV) and thyroid cancer in a cohort from southern China (Yu et al.).

The articles leaded by Prof. Guangwen Cao investigated urban-rural disparity in cancer burden during 2002–2015 in Shanghai, China. They concluded that female breast cancer and CRC occurred more frequently in urban than in rural populations, while the mortality of female breast cancer significantly declined in urban and rural areas. For all cancers combined, the 5-year survival estimate was higher in urban than in rural areas. These findings provide evidence to optimize the strategy for cancer control and prevention in Shanghai, China (Li et al.).

Using the Kailuan men cohort study with overall 104,825 men participating in the health checkup during 2006–2015, Prof. Jie He' group from National Cancer Center found that the U-shaped association between waist circumference and liver cancer risk tended to be strengthened among men with hepatitis B surface antigen (HBsAg) negativity, suggesting waist circumference might be an independent predictor of liver cancer risk in men, especially for those with HBsAg negativity (Wei et al.). Another study from Prof. Jie He' group found the diagnostic yield of ultrasound screening for breast cancer in high-risk population was satisfactory by analyzing 72,250 women with high-risk for breast cancer derived from the Cancer Screening Program in Urban China during 2012–2016 (Wang et al.).

Using a multicenter case-control study, Mai et al. from Hongkong found consumption of milk across life periods was associated with lower risks of nasopharyngeal carcinoma (NPC), which might have important implications for dairy product consumption and prevention of NPC. A systematic review from Prof. Zhijun Dai's group found a strong association between incidence and mortality risk of cancers and concentration of Hexavalent chromium [Cr(VI)] in the air and the exposure time (Deng et al.).

He et al. showed a successful application of electronic health record (e-HR) system in cancer prevention and control in Minhang district of Shanghai, China. Pingping et al. reported the first population-based study investigating epidemiology of sarcomas in Shanghai according to anatomic site and histologic type. Men et al. provided the first geographically representative epidemiological study of postoperative radiotherapy (PORT) in non-small cell lung cancer (NSCLC) patients in China, showing a declined trend of PORT use from 2005 to 2014. Lu et al. found the 7th edition of the TNM classification for esophageal carcinoma is poorly recognized and understood in central and southern China, which might contribute to the relatively low rate of appropriate perioperative procedures applied for esophageal cancer patients.

## Author Contributions

TC drafted the manuscript. TC, XS, HL, and JJ revised the manuscript and approved the submission. TC and JJ take responsibility as the corresponding authors.

### Conflict of Interest

The authors declare that the research was conducted in the absence of any commercial or financial relationships that could be construed as a potential conflict of interest.

